# Childhood neurodivergent traits, inflammation and chronic disabling fatigue in adolescence: a longitudinal case–control study

**DOI:** 10.1136/bmjopen-2024-084203

**Published:** 2024-07-02

**Authors:** Lisa Quadt, Jenny Csecs, Rod Bond, Neil A Harrison, Hugo D Critchley, Kevin A Davies, Jessica Eccles

**Affiliations:** 1 Department of Clinical Neuroscience, Brighton and Sussex Medical School, Brighton, UK; 2 Berkshire Healthcare NHS Foundation Trust, Bracknell, Bracknell Forest, UK; 3 School of Psychology, University of Sussex, Brighton, UK; 4 Cardiff University Brain Research Imaging Centre, Cardiff University, Cardiff, UK; 5 Department of Clinical and Experimental Medicine, Brighton and Sussex Medical School, Brighton, UK; 6 Sussex Partnership NHS Foundation Trust, Worthing, UK

**Keywords:** adolescents, developmental neurology & neurodisability, fatigue

## Abstract

**Abstract:**

**Objectives:**

To test whether inflammatory processes link the expression of childhood neurodivergent traits to chronic disabling fatigue in adolescence.

**Design:**

Longitudinal case–control study.

**Setting:**

We analysed data from The Avon Longitudinal Study of Parents and Children (ALSPAC).

**Participants:**

8115 and 8036 children of the ALSPAC cohort at ages 7 and 9 years, respectively, 4563 of whom also completed self-report measures at age 18 years.

**Primary and secondary outcome measures:**

We assessed if children scoring above screening threshold for autism/attention deficit hyperactivity disorder (ADHD) at ages 7 and 9 years had increased risk of chronic disabling fatigue at age 18 years, computing ORs and CIs for effects using binary logistic regression. Mediation analyses were conducted to test if an inflammatory marker (interleukin 6 (IL-6)) at age 9 years linked neurodivergent traits to chronic disabling fatigue at age 18 years.

**Results:**

Children with neurodivergent traits at ages 7 and 9 years were two times as likely to experience chronic disabling fatigue at age 18 years (likely ADHD OR=2.18 (95% CI=1.33 to 3.56); p=0.002; likely autism OR=1.78 (95% CI=1.17 to 2.72); p=0.004). Levels of IL-6 at age 9 were associated with chronic disabling fatigue at age 18 (OR=1.54 (95% CI=1.13 to 2.11); p=0.006). Inflammation at age 9 years mediated effects of neurodivergent traits on chronic disabling fatigue (indirect effect via IL-6: ADHD b=1.08 (95% CI=1.01 to 1.15); autism b=1.06; (95% CI=1.03 to 1.10)). All effects remained significant when controlling for the presence of depressive symptoms.

**Conclusions:**

Our results indicate higher risk of chronic disabling fatigue for children with neurodivergent traits, likely linked to higher levels of inflammation. The implementation of transdiagnostic screening criteria to inform support strategies to counteract risk early in life is recommended.

**STRENGTHS AND LIMITATIONS OF THIS STUDY**

- A large, longitudinal data set was used to assess potential mechanisms underlying the association between childhood neurodivergent traits and chronic disabling fatigue.
- Sample size was too small for subgroup analysis to detect risk according to sex.
- Neither neurodivergent traits nor chronic disabling fatigue was clinically assessed, but based on self-report and parent report.

## Introduction

 There is an increasing interest in the aetiology and maintenance of chronic fatigue, including experiences of fatigue symptoms associated with medical conditions, and chronic fatigue syndromes such as myalgic encephalomyelitis (ME/CFS).^1^ Fatigue has particularly come into focus in the wake of the COVID-19 pandemic and the emergence of Long COVID, where prolonged fatigue is a major symptom in both children^2^ and adults.^3 4^ However, there is still substantial debate about which risk factors may make certain groups more susceptible to developing chronic fatigue throughout the lifespan.

‘Neurodivergence’ describes neurodevelopmental conditions such as autism and attention deficit hyperactivity disorder (ADHD) and supersedes the traditional, deficit-based view of these conditions.^5^ In this article, we align our language with the preferences of the neurodivergent community, including using identity-first language where appropriate and avoiding ableist and pathologising terms.^6^,^8^ The prevalence for autism is estimated at 1%^9^ and between 2.7% and 5.4% for ADHD.^10^ However, recent reports estimate that at least 10%–15% of the population is neurodivergent.^11 12^ Conservative diagnostic criteria perhaps contribute to underdiagnosis of individuals assigned female at birth^13^ and ethnic minorities,^14^ making accurate prevalence estimates difficult.

Neurodivergent individuals are likely at higher risk of experiencing chronic fatigue. A substantial evidence base indicates that neurodivergence frequently co-occurs with mental and physical health conditions. These include chronic pain (eg, fibromyalgia),^15^ conditions related to hereditary connective tissue disorders (eg, Ehlers-Danlos syndrome/hypermobility spectrum disorder),^16^ digestive and gut issues,^17 18^ frequent dental complaints,^19^ and common psychiatric conditions, such as anxiety and depression.^20 21^ Increasingly, it is suggested that ADHD and chronic fatigue share transdiagnostic clinical features, such as executive dysfunction in personal, occupational and social domains,^22^,^24^ atypical attention and sleep patterns^25 26^ and increased risks for co-occurring health conditions.^27 28^ Previous studies have investigated the overlap between ADHD and (chronic) fatigue, with a focus on symptomatic overlap,^29^,^32^ and neurochemical mechanisms.^33^ A study comparing patients with ME/CFS, autistic participants, and a comparison group without diagnoses of these conditions, found that ME/CFS patients did not score higher on an autism screening tool than the comparison group.^34^ Recent qualitative research may suggest symptomatic overlap between (chronic) fatigue and ‘autistic burnout’,^35 36^ and one empirical study found increased rates of ‘central sensitivity syndromes’, including ME/CFS, in autistic adults.^37^

Research into the potential co-occurrence of neurodivergence and chronic fatigue has focused hitherto on adult populations, although neurodivergent children are likely also at higher risk. Crucially, there is evidence that children with chronic fatigue present differently to adults,^38^ and it is important to differentiate the experience of (chronic) fatigue from fatigue syndromes, such as ME/CFS. One classification criterion for fatigue conditions is the duration of fatigue, ranging from acute fatigue (<1 month), prolonged (>1 month, <6 months) to chronic fatigue (>6 months).^39^ ME/CFS, in contrast to acute and prolonged fatigue, has very specific diagnostic criteria, and is characterised by unrefreshing sleep and rest, chronic widespread pain, memory, attentional and cognitive dysfunction.^40^ In many countries, a medical diagnosis of ME/CFS requires the presence of postexertional malaise (PEM) as a cardinal feature.^41^

Despite its evident detrimental impact on quality of life, prolonged or chronic fatigue as a medical symptom is frequently described as an ‘unexplained’ symptom,^42^ and patients regularly report being dismissed about their experience. However, there is robust evidence^43^ for a variety of biological mechanisms, including inflammation in the pathobiology of fatigue.^44^ Dysregulated immunological and inflammatory processes are likely implicated in the development of fatigue symptomatology, including persisting production of proinflammatory cytokines after viral or bacterial infections, or other stressors.^40 44^ In chronic fatigue syndrome, mitochondrial, T cells, B cells and natural killer cells dysfunction and dysregulated circulating cytokines in combination with dysfunctional activation of the hypothalamic–pituitary–adrenal axis may be involved in the onset of chronic fatigue.^45^ A recent case–control study with Long COVID patients shows that exercise resulted in attenuated mitochondrial enzyme activity, reduced T-cell response and tissue infiltration in skeletal muscle, strongly indicating that local and systemic metabolic disturbances underlie PEM.^46^ Aberrant immune responses and increased inflammation may underlie fatigue in the general population, where increased interleukin 6 (IL-6) is associated with so-called ‘sickness behaviours’.^47^ Sickness behaviours are a normal reaction to signals from peripheral inflammatory cytokines, and include fatigue, anhedonia and social withdrawal.^48^ The production of the proinflammatory cytokine IL-6 is part of an immediate immune response to homeostatic threats, and its synthesis usually ceases when homeostasis is restored. When IL-6 synthesis is dysregulated and persists, however, fatigue may persist, making IL-6 a target for clinical and interventional research.^49^

Both neuroinflammatory and peripheral inflammatory dysfunction may further provide a mechanistic link between neurodivergence and chronic fatigue. Dysregulation of peripheral inflammatory cytokines is documented for autism^50^ and ADHD,^51^ where higher levels of IL-6 are found in both populations. It is also suggested that neuroinflammation links ADHD with pain disorders.^52^ Additionally, increased inflammation mediated by mast cell activation is proposed to play a mechanistic role in the neurodevelopment of autism^53 54^ and ADHD.^55^ Mast cells are blood cells reacting to immune stressors, often described as the ‘immune gate to the brain’.^56^ Mast cell activation syndrome (MCAS) describes a range of mast cell disorders, typically characterised by overactivation of mast cell mediators in response to internal or external triggers. Symptoms range widely from mild allergic reactions to life-threatening anaphylaxis.^57^ There is furthermore increasing evidence to suggest a mechanistic and symptomatic overlap between variant connective tissue, MCAS and chronic fatigue.^58^ These conditions are over-represented in neurodivergent populations,^16^ strengthening a potential role of aberrant inflammatory processes involved in chronic fatigue experienced by neurodivergent individuals.

We aimed to investigate the relationship between increased experiences of chronic fatigue and neurodivergent traits in a longitudinal birth cohort. We hypothesised a neurodevelopmental pathway from childhood neurodivergent (autism, ADHD) traits to experiencing chronic disabling fatigue in adolescence and tested whether increased peripherally circulating inflammatory markers (IL-6) impacted on this relationship. We chose IL-6 as previous studies showed increased levels in both autistic^50^ and ADHD populations.^51^

## Methods

### Study design and participants

We used data from The Avon Longitudinal Study of Parents and Children (ALSPAC), a birth cohort that records data of 14 541 live births from women in Avon County in southwest England between April 1991 and December 1992.^59^ Parents and children regularly completed postal questionnaires about a comprehensive number of aspects of their child’s and their own health and development from birth. Children attended annual assessment clinics from the age of 7 years, during which a series of physiological tests and face-to-face interviews was conducted.

**Figure 1:**
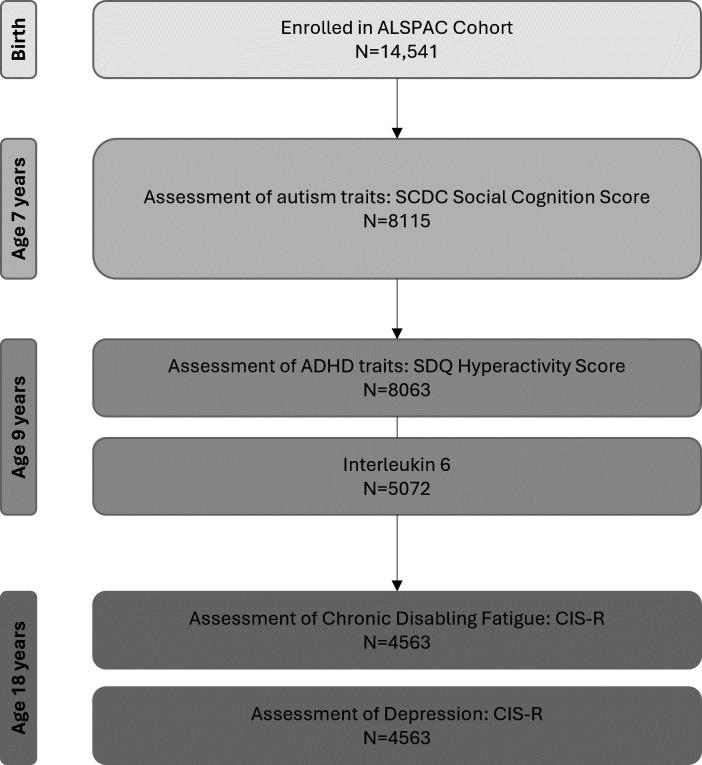
Participant Flow and available data at each timepoint. ADHD, attention deficit hyperactivity disorder; ALSPAC, Avon Longitudinal Study of Parents and Children; CIS-R, Clinician Interview Schedule Revised; SCDC, Social and Communication Disorders Checklist; SDQ, Strengths and Difficulties Questionnaire.

Data from assessments at ages 7, 9 and 18 years were used for this study, as variables of interest were tested at these timepoints (figure 1). At age 7 years, parents and carers of 8115 children filled out the Social and Communication Disorders Checklist (SCDC),^60^ of which the Social Cognition Score was used as an indication of autistic traits. At age 9 years, parents or carers of 8063 children completed the Strengths and Difficulties Questionnaire (SDQ),^61^ of which we used the Hyperactivity Score to get an estimate of ADHD traits. 5072 of these children also attended a clinic where IL-6 was assessed. At age 18 years, the same participants were asked to complete the Clinical Interview Schedule Revised (CIS-R),^62^ 4563 respondents provided data.

### Outcomes

#### Childhood neurodivergent traits at ages 7 and 9 years

Neurodivergent traits were measured at ages 7 and 9 through parent report using the SCDC^60^ for autistic traits and the SDQ^61^ Hyperactivity subsection for ADHD traits. The SDQ entails five subscales (hyperactivity, conduct problems, emotional symptoms, peer problems and prosocial behaviour), of which we used the hyperactivity subscale as an index of ADHD traits. Scores range between 0 and 40, with higher scores indicating greater difficulties. In the case of missing data at the item level, scores were prorated. The SCDC is a brief, 12-item questionnaire assessing autistic traits. Scores range between 0 and 24, with higher scores indicating higher likelihood of meeting diagnostic criteria for autism. The SCDC and SDQ were completed by parents when children were 7 years and 7 months, and 9 years and 7 months old, respectively.

We calculated binary variables for presence/absence of likely autism and ADHD using the recommended cut-off scores of ≥8 for likely presence of ADHD^63^ and ≥9 for likely presence of autism.^64^

#### IL-6 at age 9 years

Blood draws for serum IL-6 concentrations were taken during assessment clinic visits from non-fasting participants. Blood draws were taken either in the morning or early afternoon, depending on whether the child attended a morning or afternoon clinic. We excluded cases that had reported very recent or current infections at the time of the blood draw to avoid confounding by higher inflammation markers.

#### Chronic disabling fatigue and depression at age 18 years

Chronic disabling fatigue and depression were assessed at age 18 years using the self-administered computerised version of the CIS-R.^62^ We included a measure of depression to test for potential confounding effects of depressed mood. Participants were classified as experiencing ‘chronic disabling fatigue’ as defined by Collin *et al*^65^ if they met the following criteria: (1) they had been lacking energy and getting tired during the last month, (2) they responded ‘yes’ to more than two of the following four items: (a) feeling tired or lacking energy for 4 days within the past 7 days; (b) feeling tired of lacking energy for more than 3 hours in total on any day in the past 7 days; (c) feeling so tired or lacking in energy that they had to push themselves to get things done on one or more occasions in the past 7 days; (d) feeling tired or lacking energy when doing things they enjoy in the past 7 days, (3) their fatigue lasted longer than 6 months, (4) their fatigue was not explained by exercise or medication, (5) their fatigue was not alleviated by rest and (6) their fatigue was worse after exercise.^65^ We use the term ‘chronic disabling fatigue’ instead of ME/CFS to indicate that this was based on self-report instead of a clinical assessment by a physician following a diagnosis of ME/CFS.^66^

### Statistical analyses

Statistical analyses were conducted using IBM SPSS Statistics V.26 and MPlus V.8.^67^ We tested whether scoring above cut-off for autism and ADHD traits at ages 7 and 9 years predicted subsequent chronic disabling fatigue at age 18 years by using logistic regression to calculate ORs and 95% CIs for chronic disabling fatigue separately for ADHD and autistic traits. We used logistic regression to explore the association between IL-6 at age 9 years and subsequent chronic disabling fatigue at age 18. We repeated these analyses controlling for depression.

As a sensitivity analysis, we repeated these analyses again using continuous SDQ and SCDC scores to test whether we would get similar results.

We also performed two separate mediation analyses^68^ with chronic disabling fatigue at 18 years as binary outcome (present/absent), or SCDC or SDQ scores (continuous) at 7 and 9 years as predictors, and levels of IL-6 at 9 years as the potential mediating variable. We repeated mediation analyses to control for CIS-R Depression Score. For the mediation analysis, we used probit regression with full information maximum likelihood (MPlus estimator command *ML* with Monte Carlo integration), applying bootstrapping (n=2000) with the generation of 95% bias-corrected bootstrap CIs to test inferentially for direct and indirect mediation effects.

We applied full information maximum likelihood estimation,^69^ where a likelihood function calculates the relationship between a probability estimate based on observed data from variables in the respective analytical model and different estimate values. Full information maximum likelihood estimation chooses parameter estimates that maximise this likelihood function based on complete cases, thereby providing robust parameter estimates, despite missing data.^70^

### Patient and public involvement

This research was motivated by ongoing patient and public involvement work, where neurodivergent young people, and parents and caregivers of neurodivergent children and young people often express their concerns about physical and mental health conditions. Chronic fatigue and fatiguing conditions in adolescence are specific concerns that are exacerbated by the COVID-19 pandemic and the emergence of Long COVID. In conversation with patients, parents and caregivers, it was often expressed that postviral or postinflammatory fatigue was experienced but dismissed and underappreciated by healthcare professionals. Two patient representatives commented on this manuscript. Our patient and public involvement members will support the dissemination of our findings among public stakeholders in an accessible manner, and the author team includes neurodivergent individuals.

## Results

### Frequency of chronic disabling fatigue and neurodivergent traits

Table 1 shows frequency of scoring above threshold for autism and ADHD traits at ages 7 and 9 years, and for chronic disabling fatigue at age 18 years.

**Table 1:** Frequency of neurodivergent traits and chronic disabling fatigue

	Data available for ages 7 and 9 years	Above cut-off	Data available for age 18 years	Above cut-off
Autism	8115	629 (7.8)		
ADHD	8063	373 (4.6)		
Chronic disabling fatigue			4563	367 (8.0)

Data are n (%) or n/N (%) unless otherwise specified. N=14 541 originally enrolled in ALSPACAvon Longitudinal Study of Parents and Children cohort. ADHD cut-off =≥9 on hyperactivity subscale on SDQStrengths and Difficulties Questionnaire. Autism cut-off =≥8 on SCDCSocial and Communication Disorders Checklist.

ADHDattention deficit hyperactivity disorder

### Risk of chronic disabling fatigue at age 18 years according to neurodivergent traits at ages 7 and 9 years

**Figure 2:**
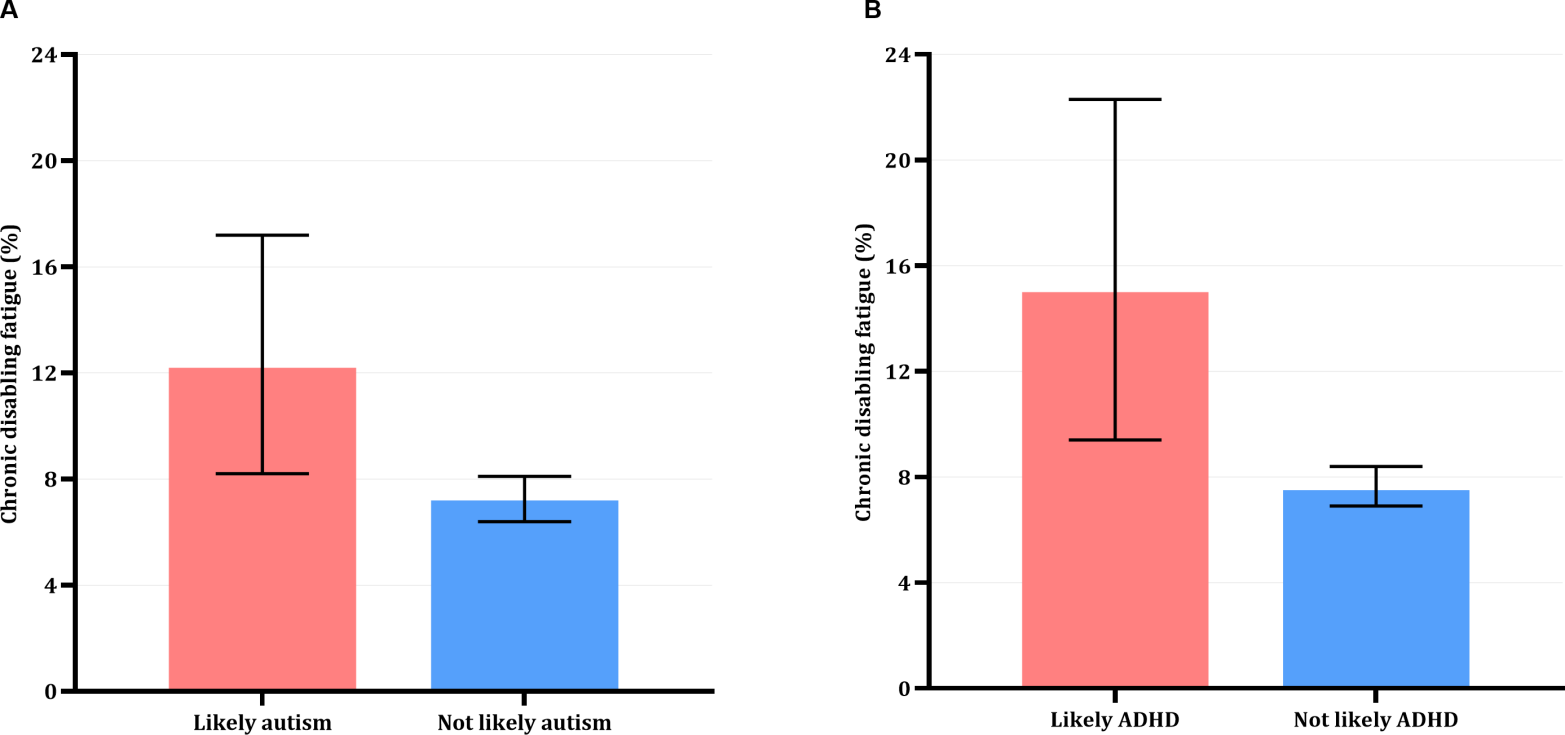
Frequency of chronic disabling fatigue in percent at age 18 years for children with likely neurodivergence (likely autism (A); likely ADHD (B); red bars) and without likely neurodivergence (blue bars). Error bars are 95% CIs.

Children who met criteria for likely autism or ADHD showed higher frequency of chronic disabling fatigue at age 18 years in comparison to children who did not meet criteria (figure 2).

Children who showed an indication of autism at age 7 years were almost two times as likely (OR=1.78 (95% CI=1.17 to 2.72; p=0.008)) to experience chronic disabling fatigue at age 18 years. Children who scored above threshold for ADHD at age 9 years were two times as likely (OR=2.18 (95% CI=1.33 to 3.56; p=0.002)) to experience chronic disabling fatigue at age 18 years. These effects remained significant when controlling for depression (autism: OR=1.65 (95% CI=1.04 to 2.61; p=0.34); ADHD: OR=1.88 (95% CI=1.09 to 3.22; p=0.022)).

Sensitivity analyses showed that when using continuous SCDC and SDQ scores, differential relationships remained and likelihood of experiencing chronic disabling fatigue at age 18 years increased with higher SCDC (OR=1.06 (95% CI=1.03 to 1.09; p<0.001)) and SDQ (OR=1.08 (95% CI=1.03 to 1.14; p=0.003)). These effects remained significant when controlling for depression (SDQ: OR=1.07 (95% CI=1.02 to 1.14; p=0.013); SCDC: OR=1.05 (95% CI=1.02 to 1.09; p=0.003)).

### Association between inflammation at age 9 years and subsequent presence of chronic disabling fatigue at age 18 years

**Figure 3:**
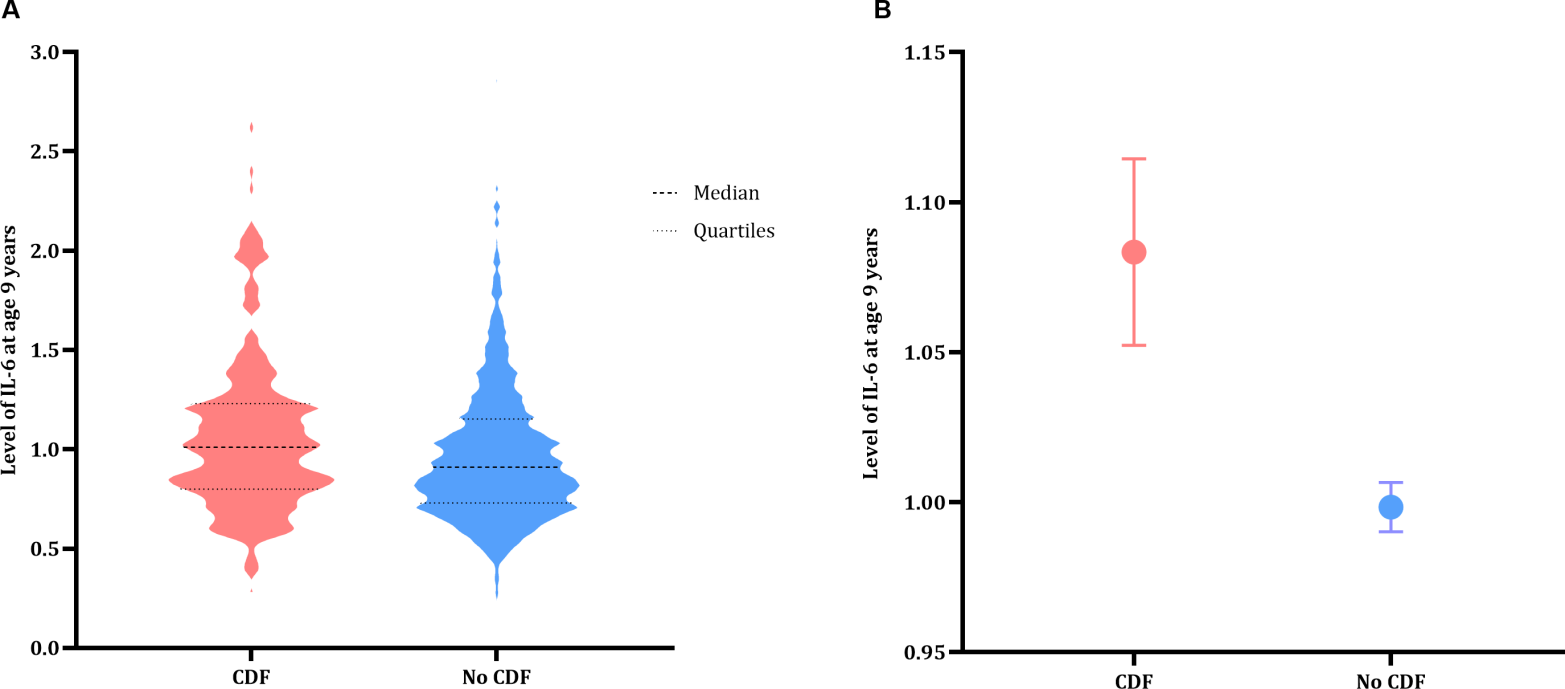
Group comparison of participants at age 18 years old who scored above threshold for chronic disabling fatigue (CDF) (CDF; red) and who did not (no CDF; blue). (A) A violin plot displaying the distribution of IL-6 levels at age 9 years old. (B) The mean IL-6 values and SEM in each group at age 9 years old. CDF, chronic disabling fatigue; IL-6, interleukin 6.

IL-6 levels at age 9 years were significantly associated with a higher likelihood of chronic disabling fatigue at age 18 years (OR=1.54 (95% CI=1.13 to 2.11); p=0.006; figure 3). This indicates a 1.5 times higher likelihood of experiencing chronic disabling fatigue at age 18 years per unit increase of IL-6 levels at age 9 years. This remained significant when controlling for depression (OR=1.59 (95% CI=1.34 to 2.22); p=0.006).

### Mediation analysis

**Figure 4:**
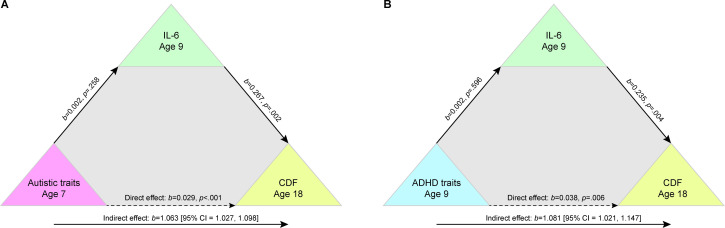
Two separate mediation analyses showing significant indirect effects of autistic traits at age 7 years (Social and Communication Disorders Checklist scores (A)) and attention deficit hyperactivity disorder traits (Strengths and Difficulties Questionnaire Hyperactivity subscale scores (B)) at age 9 years on chronic disabling fatigue at age 18 through IL-6. ADHD, attention deficit hyperactivity disorder; CDF, chronic disabling fatigue; IL-6, interleukin 6.

Both mediation models showed that continuous scores of neurodivergent traits (SCDC at age 7 years/SDQ at age 9 years) and IL-6 at age 9 years were significantly associated with presence of chronic disabling fatigue at age 18 years. This was demonstrated by significant indirect effects of IL-6 on the relationship between autistic traits and chronic disabling fatigue (b=1.06 (95% CI=1.03 to 1.10); figure 4A), and on the relationship between ADHD traits and chronic disabling fatigue (b=1.08 (95% CI=1.02 to 1.15); figure 4B). This indicates that per unit increase in IL-6, the odds that children with higher neurodivergent traits at ages seven and 9 years would experience chronic disabling fatigue at age 18 years increased by 1.06 and 1.08.

Both effects remained significant after controlling for levels of depression at age 18 years (autistic traits: b=1.06 (95% CI=1.02 to 1.10); ADHD traits: b=1.08 (95% CI=1.01 to 1.16)).

## Discussion

Using a large birth cohort, we found a link between neurodivergent traits in childhood and increased frequency of experiencing chronic disabling fatigue in adolescence, mediated by levels of childhood inflammation. We found that children who scored above cut-off on parent-reported autism and ADHD measures at ages 7 and 9 years were approximately two times as likely to meet self-reported criteria for chronic disabling fatigue at age 18 years. Increased inflammation, as indicated by IL-6 levels at age 9 years, provides a potential mechanistic explanation for this link, even when controlling for depression.

Adding to previous research indicating that there is a transdiagnostic overlap between (chronic) fatigue and ADHD, we provide evidence that also extends to autism, where little research has hitherto shown such a relationship. In line with findings that fatigue conditions may be inflammation-related,^47^ heightened IL-6 in childhood was found to be a potential explanatory link between childhood neurodivergent traits and chronic disabling fatigue.

There is ample evidence of increased inflammation in neurodivergent children and young people. Most prominently, digestive and gut issues have been associated with increased local and systemic inflammation,^71^ but frequent dental issues may also be connected with inflammatory conditions.^72^ Additionally, higher biopsychosocial stress in neurodivergent children perhaps accounts for increased levels of inflammation and subsequent experience of chronic disabling fatigue. In the ALSPAC cohort, blood samples from which IL-6 levels were detected were drawn at a clinical appointment. It is possible that this session and the blood draw itself introduced stress to young participants, especially those with higher neurodivergent traits, which may have impacted on IL-6 levels.

Research shows that neurodivergent children experience higher levels of loneliness,^73 74^ and are more likely to experience bullying.^75^ Neurodivergent children are also more vulnerable to experience pain.^76^,^78^ Different reasons are suggested in the literature, including differences in pain perception,^79^ dopaminergic dysregulation,^52^ and increased prevalence of hereditary connective tissue disorders, such as (hypermobile) Ehlers-Danlos syndrome.^80^ Variant connective tissue is linked to increased pain^16^ and a higher risk for psychiatric disorders in adolescents.^81^ Neurodivergence and chronic fatigue are conditions with complex individual neurodevelopmental pathways. It is therefore likely that inflammation is not the only mediating or moderating factor that plays into the over-representation of (chronic) fatigue in neurodivergent individuals. Additional mechanistic insights are needed to disentangle the intricacies of this relationship. It is likely that a convergence of exogenous factors in a vulnerable body increases the risk of experiencing chronic fatigue in neurodivergent young people, as evidence from our results suggests.

### Strengths and limitations

Although both SDQ and SCDC have good psychometric properties in identifying indications of ADHD and autism,^64 82^ it is important to note that they cannot replace clinical evaluations for neurodivergent conditions. Additionally, these were not child-completed questionnaires. Parents’ report on neurodivergence in their children has been shown to be biased depending on race^83^ and sex^84^ of the child and may thus be a biased resource. Autistic advocates and researchers call for using measurement tools that meaningfully reflect the autistic experience, which may not always align with their psychometric properties.^85^ Furthermore, the concept of chronic disabling fatigue was carefully conceptualised in this study, but again cannot replace clinical assessment. Future research can improve robustness by testing associations between clinically evaluated diagnoses.

Although we used a large birth cohort, we did not perform a subgroup analysis to detect risk according to sex, as sample size was too small.

ALSPAC blood sample collection clinics ran both morning and afternoon sessions. As IL-6 exhibits diurnal variation with decreasing levels throughout the day, the collection time may have impacted on IL-6 levels in participant blood samples depending on time of attendance and blood draw.

We found a rather large incidence of chronic disabling fatigue in cohort participants at age 18 years (8%). In contrast, a systematic review and meta-analysis found for ME/CFS ‘an average prevalence of 1.40%±1,57%, with a pooled prevalence of 0.39% and a meta-analysis prevalence of 0.68%’.^86^ However, chronic fatigue conditions, including ME/CFS, are likely largely underdiagnosed or misdiagnosed,^87^ with one study citing up to 91% of patients being undiagnosed or misdiagnosed.^88^ Additionally, our sample was not clinician tested for ME/CFS, perhaps also accounting for a higher number of chronic disabling fatigue.

### Conclusion

Our findings call for transdiagnostic screening practices to support health and well-being during development and adolescence. Critically, children who are suspected to be or diagnosed as neurodivergent should routinely be screened for physical and mental health concerns. Earlier integration of brain-body concerns in a holistic framework can facilitate tailored support and improve quality of life of neurodivergent individuals of all ages. We therefore call for timely access to healthcare, extra attention, and early intervention targeting health concerns at critical timepoints for neurodivergent children.

## Data Availability

All data are available on the ALSPAC data dictionary and variable search tool (http://www.bristol.ac.uk/alspac/researchers/our-data/).
