# Supplementary material for: Childhood neurodivergent traits, inflammation and chronic disabling fatigue in adolescence: a longitudinal case–control study
Source: BMJ Open. 2024 Jul 2;14(7):e084203. doi: 10.1136/bmjopen-2024-084203 (PMC11733788; doi:10.1136/bmjopen-2024-084203)
Supplement: Supplementary file 1 [file bmjopen-2024-084203.reviewer_comments.pdf]

PEER REVIEW HISTORY

BMJ Open publishes all reviews undertaken for accepted manuscripts. Reviewers are asked to complete a checklist review form (**http://bmjopen.bmj.com/site/about/resources/checklist.pdf**) and are provided with free text boxes to elaborate on their assessment. These free text comments are reproduced below.

ARTICLE DETAILS

**TITLE (PROVISIONAL)**	Childhood neurodivergent traits, inflammation, and chronic disabling fatigue in adolescence: a longitudinal case-control study
**AUTHORS**	Quadt, Lisa; Csecs, Jenny; Bond, Rod; Harrison, Neil; Critchley, Hugo; Davies, Kevin; Eccles, Jessica

VERSION 1 – REVIEW

**REVIEWER**	Takahashi, Nagahide<br>Nagoya University, Child and Adolescent Psychiatry
**REVIEW RETURNED**	07-Feb-2024

**GENERAL COMMENTS**	This is the study that investigated the possible association between neurodevelopmental disorders such as ADHD and ASD in childhood and chronic disabling fatigue in young adulthood. They used the ALSPAC cohort, which collected relatively large data (N=4563). The analysis is straightforward and the conclusion is clear. I have only a few minor comments.<br><br>Abstract<br>“All effects remained significant when controlling for levels of depression.”<br>A description such as "controlling for the presence of depressive symptoms" might be better, as the authors do not seem to include levels of depression in the analysis.<br><br>Introduction<br>The authors have focused too much on the relationship between ADHD and chronic fatigue. I understand that the evidence between ASD and chronic fatigue is sparse, but should be described as their hypothesis included both conditions as risk factors for chronic fatigue.<br><br>The authors should describe in the introduction why they chose IL-6 and not CRP as a marker of inflammation.<br><br>Results.<br>Considering recent data on the prevalence of ADHD and ASD, the prevalence of ADHD is relatively low and that of ASD was high in the present sample.<br><br>Strength and Limitation<br>“Additionally, as both the SDQ and SCDC are traditional screening measures that may be biased towards detecting autism in boys, this may have introduced a gender bias, which would give misleading findings.”<br>If the authors believe that the SDQ and SCDC may have a gender bias, how can the current results be considered misleading?

	Figure 2.<br>The reviewer does not understand what the error bars of "prevalence of chronic disabling fatigue" mean in each group.<br><br>Figure 3.<br>Figure 3B shows the mean IL-6 levels and SEM in each group at the age of 9 years. What does SEM mean?

**REVIEWER**	Kerekes, Nore<br>University West, Trollhättan, Health Sciences
**REVIEW RETURNED**	11-Feb-2024

**GENERAL COMMENTS**	Dear Authors,<br>Thank you for your comprehensive manuscript detailing the critical importance of transdiagnostic screening practices in supporting the health and well-being of individuals during development, particularly those who have neurodevelopmental disorders. Your study underscores the necessity for routine screening for both physical and mental health concerns in children suspected to be or diagnosed with neurodevelopmental disorders.<br>I believe in the importance of the integration of brain-body concerns within a holistic framework, as advocated by your research, holds significant promise in providing tailored support and enhancing the quality of life for individuals with ADHD and/or ASD across all age groups. Your emphasis on the need for earlier intervention and support aligns well with current best practices in healthcare.<br>The longitudinal nature of your study, along with the large cohort size, lends credibility to your findings. The observed associations between "likely-ADHD" and "likely-ASD" traits at age 9 and the increased risk of experiencing chronic disabling fatigue (CDF) at age 18, as well as the mediation effects of inflammation on this relationship, provide new evidence to this field of research.<br><br>Overall, you presented a nicely written and high quality work that I congratulate you to. Your study significantly contributes to our understanding of the complex interplay between neurodevelopmental disorders, inflammation, and long-term health outcomes.<br><br>I have carefully assessed both major and minor aspects of your work in my review, aiming to enhance its quality and comprehensibility. My intention is to provide constructive feedback to contribute to the improvement of this valuable and significant piece of research. Please find them enclosed.

VERSION 1 – AUTHOR RESPONSE

Reviewer 1

#	Reviewer comment	Response
1	This is the study that investigated the possible association between neurodevelopmental disorders such as ADHD and ASD in childhood and chronic disabling fatigue in young adulthood. They used the ALSPAC cohort, which collected relatively large data (N=4563). The analysis is straightforward and the	*Thank you for your positive evaluation of our manuscript!*

	conclusion is clear. I have only a few minor comments.	
2	"All effects remained significant when controlling for levels of depression."<br>A description such as "controlling for the presence of depressive symptoms" might be better, as the authors do not seem to include levels of depression in the analysis.	*We have changed this:*<br><br>All effects remained significant when controlling for the presence of depressive symptoms.
3	The authors have focused too much on the relationship between ADHD and chronic fatigue. I understand that the evidence between ASD and chronic fatigue is sparse, but should be described as their hypothesis included both conditions as risk factors for chronic fatigue.	*We have included more information in the introduction:*<br><br>Increasingly it is suggested that ADHD and chronic fatigue share transdiagnostic clinical features, such as executive dysfunction in personal, occupational, and social domains,[24-26] atypical attention and sleep patterns,[27 28] and increased risks for co-occurring health conditions.[29 30] Previous studies have investigated the overlap between ADHD and (chronic) fatigue, with a focus on symptomatic overlap,[31-34] and neurochemical mechanisms.[35] . In a study comparing patients with ME/CFS, autistic participants, and a comparison group without diagnoses of these conditions found that ME/CFS patients did not score higher on an autism screening tool than the comparison group.[36] Recent qualitative research may suggest symptomatic overlap between (chronic) fatigue and 'autistic burnout',[37 38] and one empirical study found increased rates of 'central sensitivity syndromes', including ME/CFS, in autistic adults.[39]
4	The authors should describe in the introduction why they chose IL-6 and not CRP as a marker of inflammation.	*We have included this in the introduction:*<br><br>We chose IL-6 as previous studies showed increased levels in both autistic[52] and ADHD populations.[53]
5	Considering recent data on the prevalence of ADHD and ASD, the prevalence of ADHD is relatively low and that of ASD was high in the present sample.	*We have included recent estimates of autism and ADHD prevalence in the introduction, and provided reasons for the likely underestimation of neurodivergence:*<br><br>The prevalence for autism and ADHD is estimated at 1%[9] and between 2.7-5.4% for ADHD[10] respectively. However, recent reports estimate that at least 10-15% of the population is neurodivergent.[11 12] Conservative diagnostic criteria perhaps contribute to underdiagnosis of individuals assigned female at birth,[13] and ethnic minorities,[14] making accurate prevalence estimates difficult.
6	"Additionally, as both the SDQ and SCDC are traditional screening measures that may be biased towards detecting autism in boys, this may have introduced a gender bias, which would give misleading findings."<br>If the authors believe that the SDQ and SCDC	*We have taken this statement out of the discussion.*

	may have a gender bias, how can the current results be considered misleading?	
7	The reviewer does not understand what the error bars of "prevalence of chronic disabling fatigue" mean in each group.	*We have clarified this in the caption:*<br><br>Figure 2: Frequency of chronic disabling fatigue in % at age 18 for children with likely neurodivergence (likely ADHD 2A; likely Autism 2B; red bars) and without likely neurodivergence (blue bars). Error bars are 95% confidence intervals.
8	Figure 3B shows the mean IL-6 levels and SEM in each group at the age of 9 years. What does SEM mean?	*We have clarified this in the caption:*<br><br>Figure 3: Group comparison of participants at age 18 years old who scored above threshold for chronic disabling fatigue (CDF; red) and who did not (No CDF; blue). Figure 3A shows a violin plot displaying the distribution of IL-6 levels at age 9 years old. Figure 3B shows the mean IL-6 values and standard error of the mean (SEM) in each group at age 9 years old. CDF=chronic disabling fatigue.

Reviewer 2

#	Reviewer comment	Response
1	The utilization of ADHD and ”autism” as categorical measures raises some concerns, given that they are based on parent-reported surveys. The category of ADHD was established using a cutoff on the Hyperactivity subdomain of SDQ. Although it was validated against ADHD in the reference article (ref nr 45), its sensitivity was measured at 0.45, with an AUC value of 0.84. Similarly, the category of "autism" was derived from a cutoff on SCDC, which was validated against autism spectrum disorder (ASD)(and not autism) (ref nr 46). Throughout the study, it is important for the authors to discern when to refer to traits and when to discuss categorical disorders. Questioning the necessity of employing cutoffs and creating categorical variables is warranted, particularly when observing that scores on SDQ and SCDC consistently exhibit a significant association with the binary outcome of CDF. This observation aligns more closely with contemporary clinical practices and thinking. Emphasizing traits over rigid diagnostic categories allows for a more adaptable, precise, and person-centered approach to understanding, researching, and supporting individuals with neurodevelopmental disorders. This approach also enables the utilization of findings from research conducted over the past two decades, acknowledging the complexity of these disorders and facilitating tailored interventions to meet	*Thank you for this thoughtful response.*<br><br>*We align our language with the preferences of the autistic and ADHD community, who largely prefer the term “autism” over “ASD” and prefer identity-first language (e.g., “autistic person” instead of “person with ASD”). We have clarified this in the introduction:*<br><br>‘Neurodivergence’ describes neurodevelopmental conditions such as autism and attention deficit hyperactivity disorder (ADHD) and supersedes the traditional, deficit-based view of these conditions. [5] In this article, we align our language with the preferences of the neurodivergent community, including using identity-first language where appropriate and avoiding ableist and pathologizing terms.[6-8]<br><br>*We agree that it is important to include continuous scores, and have added a sensitivity analysis to test whether our results still hold when we repeat our analysis with continuous SDQ and SCDC scores. We describe this in the methods and results:*<br><br>As a sensitivity analysis, we repeated these analyses again using continuous SDQ and SCDC scores to test whether we would get similar results.<br><br>Sensitivity analyses showed that when using continuous SCDC and SDQ scores, differential relationships remained and likelihood of experiencing chronic disabling fatigue at age 18

	individuals' specific needs. However, if the authors decide to retain the categorical variables, I would advise a more humble approach when working with the scores obtained from parent-rated instruments. The authors correctly utilized the expression "LikelyADHD" and "Likely-autism" in Figure 1 on page 22. It should be noted that "Likely-ASD" would be a more appropriate term. I recommend maintaining consistency by using these expressions consistently throughout the text as well.	years increased with higher SCDC (OR=1.06 [95% CI=1.03, 1.09]) and SDQ (OR=1.08 [95% CI=1.03, 1.14]). These effects remained significant when controlling for depression (SDQ: OR=1.07 [95% CI=1.02, 1.14]; SCDC: OR=1.05 [95% CI=1.02, 1.09]).<br><br>*We furthermore checked the manuscript for language and changed text to "likely ADHD", "likely autism", and "neurodivergent traits" where appropriate, and changed the title to*<br><br>Childhood neurodivergent traits, inflammation and later chronic fatigue in adolescence: a longitudinal case-control study
2	Another significant concern I have pertains to the inconsistent use of the concepts "inflammation" and "neuroinflammation" throughout the study. While some studies explore the relationship between both inflammation and neuroinflammation with psychiatric disorders, it is crucial to distinguish between these two inflammatory processes. Inflammation denotes a bodywide process that can affect ADHD, for instance, through systemic changes, whereas neuroinflammation specifically targets the nervous system and can directly impact brain functions associated with ADHD. It's plausible that systemic inflammation could exacerbate neuroinflammatory processes. However, including neuroinflammation in this study is inappropriate because while IL-6 levels in the blood commonly serve as indicators of systemic inflammation, they do not directly gauge neuroinflammation. To assess neuroinflammation, researchers typically rely on more direct methods such as neuroimaging techniques (e.g., PET scans using markers binding to activated microglia) or analyzing cerebrospinal fluid for inflammatory markers, or measuring clinically central sensitization. Therefore, I advise the authors to review their work, eliminate sections concerning neuroinflammation, and carefully reassess the interpretation of the results regarding IL-6 within the study's context.	*We do think that including information of neuroinflammation is crucial in the introduction but agree that clarification is needed with respect to the scope of our study.*<br>*We clarified that our aim was to investigate peripherally circulating inflammatory markers, and that previous research suggests a role for both inflammation and neuroinflammation in neurodivergence:*<br><br>Both neuroinflammatory and peripheral inflammatory dysfunction may further provide a mechanistic link between neurodivergence and chronic fatigue. Dysregulation of peripheral inflammatory cytokines are documented for autism[52] and ADHD,[53] where higher levels of IL-6 are found in both populations. It is also suggested that neuroinflammation links ADHD with pain disorders.[54], Additionally, increased inflammation mediated by mast cell activation is proposed to play a mechanistic role in the neurodevelopment of autism,[55 56] and ADHD.[57]<br><br>[…]<br><br>We hypothesized a neurodevelopmental pathway from childhood neurodivergent (autism, ADHD) traits to experiencing chronic fatigue in adolescence and tested whether increased peripherally circulating inflammatory markers (IL-6) impacted on this relationship.
3	In the background section, rather than providing a general description of inflammatory mechanisms, it would be intriguing to explore what is known about the predictive capacity of IL-6 in relation to psychiatric disorders, such as ADHD, ASD, depression, and anxiety. For instance: - Ting, E. Y., Yang, A. C., & Tsai, S. J. (2020). Role of Interleukin-6 in Depressive	*Please see our response to your previous comment and our addition of inflammatory mechanisms in relation to neurodivergence.*

	Disorder. International journal of molecular sciences, 21(6), 2194. https://doi.org/10.3390/ijms21062194 - Roohi, E., Jaafari, N. & Hashemian, F. On inflammatory hypothesis of depression: what is the role of IL-6 in the middle of the chaos?. J Neuroinflammation 18, 45 (2021). https://doi.org/10.1186/s12974-021-02100- 7 - Ye, Z., Kappelmann, N., Moser, S., Davey Smith, G., Burgess, S., Jones, P. B., & Khandaker, G. M. (2021). Role of inflammation in depression and anxiety: Tests for disorder specificity, linearity and potential causality of association in the UK Biobank. EClinicalMedicine, 38, 100992. https://doi.org/10.1016/j.eclinm.2021.100992 - Milaneschi, Y., Kappelmann, N., Ye, Z., Lamers, F., Moser, S., Jones, P. B., Burgess, S., Penninx, B. W. J. H., & Khandaker, G. M. (2021). Association of inflammation with depression and anxiety: evidence for symptom-specificity and potential causality from UK Biobank and NESDA cohorts. Molecular psychiatry, 26(12), 7393–7402. https://doi.org/10.1038/s41380-021-01188-w - Ferencova, N., Visnovcova, Z., Ondrejka, I., Hrtanek, I., Bujnakova, I., Kovacova, V., Macejova, A., & Tonhajzerova, I. (2023). Peripheral Inflammatory Markers in Autism Spectrum Disorder and Attention Deficit/Hyperactivity Disorder at Adolescent Age. International journal of molecular sciences, 24(14), 11710. https://doi.org/10.3390/ijms241411710 - Saghazadeh, A., Ataeinia, B., Keynejad, K., Abdolalizadeh, A., HirbodMobarakeh, A., & Rezaei, N. (2019). A meta-analysis of pro-inflammatory cytokines in autism spectrum disorders: Effects of age, gender, and latitude. Journal of psychiatric research, 115, 90–102. https://doi.org/10.1016/j.jpsychires.2019.05.019 - Darwish, A. H., Elgohary, T. M., & Nosair, N. A. (2019). Serum Interleukin-6 Level in Children With Attention-Deficit Hyperactivity Disorder (ADHD). Journal of child neurology, 34(2), 61–67. https://doi.org/10.1177/0883073818809831	
4	Given the authors' utilization of a large sample size and incorporation of bootstrapping techniques, while statistically significant results have been obtained, a more profound comprehension of the context and additional information for the reader are needed to assess practical significance. Could the authors provide effect sizes in the results to aid in	*The odds ratio and confidence interval provide an estimate of effect size in logistic and probit regression. We have clarified the implication:*<br><br>Children who showed an indication of autism at age nine years were almost twice as likely (OR=1.78 [95% CI=1.17, 2.72; *p*=0.008])to experience chronic disabling fatigue at age 18 years. Children who scored above threshold for ADHD were twice as likely (OR=2.18 [95%

	interpreting the clinical or practical relevance of their findings?	CI=1.33, 3.56; *p*=0.002]) to experience chronic disabling fatigue at age 18 years. These effects remained significant when controlling for depression (autism: OR=1.65 [95% CI=1.04, 2.61; *p*=0.34]; ADHD: OR=1.88 [95% CI=1.09, 3.22; *p*=0.022]).<br><br>[…]<br><br>IL-6 levels at age nine years were significantly associated with a higher likelihood of chronic disabling fatigue at age 18 (OR=1.54 [95% CI=1.13, 2.11]; *p*=0.006; Fig. 3) This indicates a 1.5 times higher likelihood of experiencing chronic disabling fatigue at age 18 years per unit increase of IL-6 levels at age nine years.<br><br>[…]<br><br>Both mediation models showed that continuous scores of neurodivergent traits (SDQ/SCDC) and IL-6 at age nine years were significantly associated with presence of chronic disabling fatigue at age 18 years. This was demonstrated by significant indirect effects of IL-6 on the relationship between autistic traits and chronic disabling fatigue (*b*=1.06 [95% CI=1.03, 1.10]; Figure 4a), and on the relationship between ADHD traits and chronic disabling fatigue (*b*=1.08 [95% CI=1.02, 1.15]; Figure 4b). This indicates that per unit increase in IL-6, the odds that children with higher neurodivergent traits at age nine years would experience chronic disabling fatigue at age 18 years increased by 1.06 and 1.08.
5	Please consider rewriting the presentation and interpretation of your probit regression mediation model by utilizing scale variables of ASD-trait scores and ADHD-trait scores instead of categorical variables.	*The probit regression mediation model utilized continuous SDQ/SCDC scores. We have emphasized this:*<br><br>Both mediation models showed that continuous scores of neurodivergent traits (SDQ/SCDC) and IL-6 at age nine years were significantly associated with presence of chronic disabling fatigue at age 18 years.
6	The stable prevalence of ASD worldwide is approximately 1%, whereas ADHD had a prevalence between 3% to 5% in school-aged children around the year 2000. In your study, the prevalence of ADHD is 4.6%, and that of 3 "autism" is 7.8%. How do you account for this higher prevalence of ASD in this particular birth cohort?	*We have included recent estimates of autism and ADHD prevalence in the introduction, and provided reasons for the likely underestimation of neurodivergence:*<br><br>The prevalence for autism and ADHD is estimated at 1%[9] and between 2.7-5.4% for ADHD[10] respectively. However, recent reports estimate that at least 10-15% of the population is neurodivergent.[11 12] Conservative diagnostic criteria perhaps contribute to underdiagnosis of

		individuals assigned female at birth,[13] and ethnic minorities,[14] making accurate prevalence estimates difficult.
7	I have reviewed the prevalence of CFS/ME in the study conducted by Lim, EJ., Ahn, YC., Jang, ES. et al.(https://doi.org/10.1186/s12967-020-02269-0). According to their systematic review and meta-analysis, "the total average prevalence of CFS/ME was reported to be 1.40 ± 1.57%, with a pooled prevalence of 0.39% and a meta-analysis prevalence of 0.68%". However, in your study, the prevalence of Chronic Disabling Fatigue was observed to be 8%. Could you please provide an explanation for this notable discrepancy?	*We have clarified this in the discussion:*<br><br>We found a rather large incidence of chronic disabling fatigue in cohort participants at age 18 years (8%). In contrast, a systematic review and meta-analysis found for ME/CFS "an average prevalence of 1.40 ± 1,57%, with a pooled prevalence of 0.39% and a meta-analysis prevalence of 0.68%".[88] However, chronic fatigue conditions, including ME/CFS are likely largely under- or misdiagnosed,[89] with one study citing up to 91% of patients being un- or misdiagnosed.[90] Additionally, our sample was not clinician-tested for ME/CFS, perhaps also accounting for a higher number of chronic disabling fatigue.
8	Additionally, you possess information regarding anxiety levels (from SDQ). Have you considered using this data to control for the association between chronic stress and IL-6? Furthermore, could you consider to included anxiety scores in the mediation model?	*Thank you for this consideration. To our knowledge, the SDQ does not explicitly measure anxiety symptoms, but rather emotional symptoms. To keep the study focused, we therefore decided not to include this score in our analysis.*
9	It is mentioned that "We excluded cases that had infections at the time of the blood draw," and I'm curious about how these cases were identified. IL-6 serves as a versatile and sensitive marker for both inflammation and the body's response to stress. Various stressors, whether psychological or physical (like exercise), can lead to elevated IL-6 levels. The authors introduce the concept of "Sickness behavior" and discuss the role of IL-6 and CRP in it. Is there a possibility that you also analyzed CRP or additional inflammatory markers from the blood samples? If not, why to introduce it in the background? IL-6 sensitivity to stress should be addressed in the discussion. It's worth noting that being assessed in clinics, even if it's done annually, may be interpreted as psychological stress for many of the nine-year-old children, especially those positioned at the higher end of the traits. This aligns with the authors' statement in the discussion: "Additionally, higher biopsychosocial stress in neurodivergent children perhaps accounts for increased levels of inflammation and subsequent experience of chronic disabling fatigue." Discussing these implications on your results would add depth to your analysis. See my previous comment.	*Thank you for this insightful comment. Please see our additions to the manuscript:*<br><br>We excluded cases that had reported very recent or current infections at the time of the blood draw to avoid confounding by higher inflammation markers.<br>[…]<br><br>Additionally, higher biopsychosocial stress in neurodivergent children perhaps accounts for increased levels of inflammation and subsequent experience of chronic disabling fatigue. In the ALSPAC cohort, blood samples from which IL-6 levels were detected were drawn at a clinical appointment. It is possible that this session and the blood draw itself introduced stress to young participants, especially those with higher neurodivergent traits, which may have impacted on IL-6 levels.

10	Additionally, it's worth noting that IL-6 exhibits diurnal (circadian) variation, with levels typically peaking in the morning and decreasing throughout the day. Considering this, could you provide information about the time stability of the test, or discuss this potential variability as a limitation?	*Thank you for bringing this up, we agree this is important information, and have added it to the methods and limitations:*<br><br>Blood draws were taken either in the morning or early afternoon, depending on whether the child attended a morning or afternoon clinic.<br><br>[…]<br><br>ALSPAC blood sample collection clinics ran both morning and afternoon sessions. As IL-6 exhibits diurnal variation with decreasing levels throughout the day, the collection time may have impacted on IL-6 levels in participant blood samples depending on time of attendance and blood draw.
11	Please ensure to provide the registration number for the ethical approval obtained for this study.	*Ethical approval for the ALSPAC birth cohort is complex and was ongoing for each data collection. We have added the resource where each ethical approval reference number can be found:*<br><br>Details of consent procedures and reference numbers for initial and repeated ethical approval can be found on https://www.bristol.ac.uk/alspac/researchers/research-ethics/.
12	Kindly review the manuscript for abbreviations. Only use them when they appear more than five times in the manuscript. Additionally, when abbreviations are used, ensure consistency throughout the document.	*Thank you, we have taken out abbreviations where appropriate throughout the manuscript.*
13	Please write out numbers under ten consistently throughout the manuscript.	*Thank you, we have reviewed the manuscript and changed accordingly.*
14	I find it unclear why a specific personality profile is mentioned, followed by a reference to an article about autistic spectrum conditions and borderline personality disorder (ref nr 11) in your background regarding the overlap between neurodivergence and mental health conditions. If you intend to include references to personality profiles, perhaps it would be beneficial to include a study specifically investigating this topic, e.g.: Kerekes et al (2013). ADHD, autism spectrum disorder, temperament, and character: phenotypical associations and etiology in a Swedish childhood twin study. Comprehensive psychiatry, 54(8), 1140–1147. https://doi.org/10.1016/j.comppsych.2013.05.009 On the other hand, personality profiles are distinct from personality disorders, and it's important to note that personality disorders alone do not necessarily serve as the primary or most	*We have taken this information out of the introduction.*

	accurate predictors of overall mental health conditions. Consider incorporating references such as: -Pliszka, S. R. (2015). Comorbid psychiatric disorders in children with ADHD. In R. A. Barkley (Ed.), Attention-deficit hyperactivity disorder: A handbook for diagnosis and treatment (4th ed., pp. 140–168). The Guilford Press. -van Steensel, F.J.A., Bögels, S.M. & de Bruin, E.I. Psychiatric Comorbidity in Children with Autism Spectrum Disorders: A Comparison with Children with ADHD. J Child Fam Stud 22, 368–376 (2013). https://doi.org/10.1007/s10826- 012-9587-z -Aoki et al (2022). Trajectories of Healthcare Utilization Among Children and Adolescents With Autism Spectrum Disorder and/or AttentionDeficit/Hyperactivity Disorder in Japan. Fron	
15	Page 5, line 28: Please reconsider the deletion of this information after clarifying the relevance of personality profiles in the context of neurodevelopmental disorders.	*Please see above.*
16	Page 5, line 56: Please add a reference for the definition of ME/CFS	*We have added this reference:*<br>Cortes Rivera, M., Mastronardi, C., Silva-Aldana, C., Arcos-Burgos, M., & Lidbury, B. (2019). Myalgic Encephalomyelitis/Chronic Fatigue Syndrome: A Comprehensive Review. *Diagnostics*, *9*(3), 91. https://doi.org/10.3390/diagnostics9030091
17	Discussion: On page 10, line 37, you stated that your results demonstrate a "neurodevelopmental link between neurodivergent traits in childhood and increased risk of experiencing chronic disabling fatigue in adolescence," which is incorrect. Your study has shown a statistical association between the prevalence of these conditions, but it cannot conclude that it was due to any neurobiological processes.	*We have toned this statement down:*<br><br>Using a large birth-cohort, we found a link between neurodivergent traits in childhood and increased frequency of experiencing chronic disabling fatigue in adolescence, mediated by levels of childhood inflammation.
18	It is incorrectly stated (twice) in the manuscript that the association between ADHD and chronic fatigue is "anecdotal." Please review relevant publications such as: Yamamoto T. (2022). The relationship between central fatigue and Attention Deficit/Hyperactivity Disorder of the inattentive type. Neurochemical research, 47(9), 2890–2898. https://doi.org/10.1007/s11064-022-03693-y	*Thank you for making us aware of this. We have amended the manuscript accordingly:*<br><br>Increasingly it is suggested that ADHD and chronic fatigue share transdiagnostic clinical features, such as executive dysfunction in personal, occupational, and social domains,[24-26] atypical attention and sleep patterns,[27 28] and increased risks for co-occurring health conditions.[29 30] Previous studies have investigated the overlap between ADHD and (chronic) fatigue, with a focus on symptomatic overlap,[31-34] and neurochemical mechanisms.[35] . In a study comparing patients with ME/CFS, autistic participants, and a comparison group without diagnoses of these conditions found that ME/CFS patients did not score higher on an

		autism screening tool than the comparison group.[36] Recent qualitative research may suggest symptomatic overlap between (chronic) fatigue and 'autistic burnout',[37 38] and one empirical study found increased rates of 'central sensitivity syndromes', including ME/CFS, in autistic adults.[39]<br><br>[…]<br><br>Adding to previous research indicating that there is a transdiagnostic overlap between (chronic) fatigue and ADHD, we provide evidence that also extends to autism, where little research has hitherto shown such a relationship.
19	Also, you should correctly state that previously no association between ASD and CF/ME has been found. Bileviciute-Ljungar, I., Maroti, D., & Bejerot, S. (2018). Patients with chronic fatigue syndrome do not score higher on the autism-spectrum quotient than healthy controls: Comparison with autism 5 spectrum disorder. Scandinavian journal of psychology, 59(4), 428–432. https://doi.org/10.1111/sjop.12451	*Please see our reply to your previous comment for our changes in the manuscript including this.*
20	Page 11, lines 9-13: The association between (chronic) pain and ADHD/ASD is often studied directly and not necessarily through the presence of connective tissue disorders, such as hEDS (an abbreviation that if you keep you should explain). For example, - Kerekes, N., Sanchéz-Pérez, A. M., & Landry, M. (2021). Neuroinflammation as a possible link between attention-deficit/hyperactivity disorder (ADHD) and pain. Medical hypotheses, 157, 110717. https://doi.org/10.1016/j.mehy.2021.110717 - Battison, E. A., Brown, P. C., Holley, A. L., & Wilson, A. C. (2023). Associations between Chronic Pain and Attention-Deficit Hyperactivity Disorder (ADHD) in Youth: A Scoping Review. Children, 10(1), 142. - Mundal, I., Schei, J., Lydersen, S. et al. Prevalence of chronic and multisite pain in adolescents and young adults with ADHD: a comparative study between clinical and general population samples (the HUNT study). Eur Child Adolesc Psychiatry (2023). https://doi.org/10.1007/s00787-023-02249-x - Failla, M. D., Gerdes, M. B., Williams, Z. J., Moore, D. J., & Cascio, C. J. (2020). Increased pain sensitivity and pain-related anxiety in individuals with autism. Pain reports, 5(6), e861. https://doi.org/10.1097/PR9.0000000000000861 - Lipsker, C. W., Bölte, S., Hirvikoski, T., Lekander, M., Holmström, L., & Wicksell, R. K. (2018). Prevalence of	*We have included further research on this relationship:*<br><br>Neurodivergent children are also more vulnerable to experience pain.[78-80] Different reasons are suggested in the literature, including differences in pain perception,[81] dopaminergic dysregulation,[54] and increased prevalence of hereditary connective tissue disorders, such as hypermobile Ehlers-Danlos-Syndrome.[82] Variant connective tissue is linked to increased pain[16] and a higher risk for psychiatric disorders in adolescents.[83]

	autism traits and attention-deficit hyperactivity disorder symptoms in a clinical sample of children and adolescents with chronic pain. Journal of pain research, 11, 2827–2836. https://doi.org/10.2147/JPR.S177534	
21	Page 11, line 11: The issue is not that the measures were not "childcompleted," but rather that they were not clinical evaluations, which are much more complex and include parental reports, among other assessments.	*We have changed this in the discussion and the "Strengths and Limitations of this study" summary points:*<br><br>Neither neurodivergent traits nor chronic disabling fatigue were clinically assessed, but based on self- and parent-reports<br><br>Although both SDQ and SCDC have good psychometric properties in identifying indications of ADHD and autism,[66 84] it is important to note that they cannot replace clinical evaluations for neurodivergent conditions. Additionally, these were not child-completed questionnaires. Parents' report on neurodivergence in their children has been shown to be biased depending on race[85] and sex[86] of the child, and may thus be a biased resource. Autistic advocates and researchers call for utilizing measurement tools that meaningfully reflect the autistic experience, which may not always align with their psychometric properties.[87] Furthermore, the concept of chronic disabling fatigue was carefully conceptualized in this study, but again cannot replace clinical assessment. Future research can improve robustness by testing associations between clinically evaluated diagnoses.
22	Please add figure numbers to each figure on pages 22-25. Additionally, under the flowchart, please indicate the meanings of the abbreviations.	*We have included figure numbers and added the following to the flowchart legend:*<br><br>Figure 1: Participant Flow and available data at each timepoint.<br>ADHD=attention deficit hyperactivity disorder; SDQ=Strengths and Difficulties Questionnaire; SCDC=Social and Communication Disorders Checklist; CIS-R=Clinician Interview Schedule Revised.

VERSION 2 – REVIEW

**REVIEWER**	Takahashi, Nagahide<br>Nagoya University, Child and Adolescent Psychiatry
**REVIEW RETURNED**	15-May-2024

**GENERAL COMMENTS**	The authors have ad
